# Oxygen binding and nitric oxide dioxygenase activity of cytoglobin are altered to different extents by cysteine modification

**DOI:** 10.1002/2211-5463.12230

**Published:** 2017-05-18

**Authors:** Danlei Zhou, Craig Hemann, James Boslett, Aiqin Luo, Jay L. Zweier, Xiaoping Liu

**Affiliations:** ^1^ Davis Heart and Lung Research Institute and Division of Cardiovascular MedicineDepartment of Internal MedicineCollege of MedicineThe Ohio State UniversityColumbusOHUSA; ^2^ School of Life ScienceBeijing Institute of TechnologyHaidian DistrictChina

**Keywords:** cytoglobin, disulfide bond, NO dioxygenation

## Abstract

Cytoglobin (Cygb), like other members of the globin family, is a nitric oxide (NO) dioxygenase, metabolizing NO in an oxygen (O_2_)‐dependent manner. We examined the effect of modification of cysteine sulfhydryl groups of Cygb on its O_2_ binding and NO dioxygenase activity. The two cysteine sulfhydryls of Cygb were modified to form either an intramolecular disulfide bond (Cygb_SS), thioether bonds to *N*‐ethylmaleimide (NEM; Cygb_SC), or were maintained as free SH groups (Cygb_SH). It was observed that the NO dioxygenase activity of Cygb only slightly changed (~ 25%) while the P_50_ of O_2_ binding to Cygb changed over four‐fold with these modifications. Our results suggest that it is possible to separately regulate one Cygb function (such as O_2_ binding) without largely affecting the other Cygb functions (such as its NO dioxygenase activity).

**Abbreviations**
Abs_b_: the absorbance at baseline or when time *t* approaches to infinity
Asc: ascorbate
b5: cytochrome b5
b5R: cytochrome b5 reductase
Cygb‐SC: cytoglobin with thioether bonds between a cysteine residue and *N*‐ethylmaleimide
Cygb‐SH: cytoglobin with free sulfhydryl group
Cygb‐SS: cytoglobin with intramolecular disulfide bond
DT: dithionite
DTT: dithiothreitol

Cytoglobin (Cygb), a member of the globin family, was discovered 15 years ago 1, 2, 3. Like other family members such as hemoglobin (Hb) and myoglobin (Mb), Cygb is a nitric oxide (NO) dioxygenase, which metabolizes NO in an oxygen (O_2_)‐dependent manner 4, 5. Cygb is present in splanchnic fibroblasts of various organs 6, fibroblast‐related cell lineages 7, and other cell types such as neurons 8, 9, hepatocytes 10, 11, and vascular smooth muscle cells 12. *In vivo*, Cygb may play several different roles including tumor‐suppression 13, lipid signaling of oxidative stress 14, ROS protection 15, 16, 17, NO metabolism 4, 12, nitrite reduction^18^, and regulation of vascular NO concentration 5, 19.

The Cygb monomer contains two exposed cysteine residues (Cys 38 and Cys 83) that enable Cygb to form an intramolecular disulfide bond 20, 21, 22. The formation of this intramolecular disulfide bond greatly increases the dissociation rate constant of a bound intrinsic histidine, resulting in a greater apparent binding constant of extrinsic ligands 21, 22. As a NO dioxygenase, O_2_ must bind to Cygb before it metabolizes NO. Under hypoxic conditions, Cygb with intramolecular disulfide bond (Cygb‐SS) holds more O_2_ in the form of Cygb(Fe^2+^O_2_) than Cygb with free sulfhydryl group (Cygb‐SH) and Cygb with thioether bonds between a cysteine residue and *N*‐ethylmaleimide (NEM; Cygb‐SC). However, the NO dioxygenase activity of Cygb is limited and effectively controlled by the rate of Cygb reduction 23. Thus, if the rate of Cygb reduction is not altered by modification of the sulfhydryl groups in the Cygb, the NO dioxygenase activity of Cygb may not be greatly affected even under hypoxic conditions.

It was suggested that Cygb *in vivo* is monomeric 20, 24. However, Cygb dimer and other oligomers have been reported to exist in Cygb preparations 22, 25. Although both Cygb monomer and oligomers are of interest to study, the monomer is reported to be the predominant form both *in vivo* and *in vitro* 18, 20, 24. Therefore, in this study, we focus on examination of the effect of modifying the sulfhydryl groups of the cysteine residues in monomeric Cygb on the affinity of O_2_ binding and the rate of Cygb‐mediated O_2_‐dependent NO metabolism in the presence of cellular reductants.

## Materials and methods

### Modification of the sulfhydryl groups of the cysteine residues (RSH) of Cygb

The expression and purification process of Cygb was described in our prior reports 5. As isolated Cygb was first treated with dithiothreitol (DTT, C_4_H_10_O_2_S_2_) in phosphate‐buffered saline (PBS) buffer (pH = 7.4 with 0.1 mm EDTA) on ice for about 30 min to reduce any remaining disulfide bonds in the Cygb preparation, forming a nearly homogeneous solution of Cygb in the sulfhydryl form (Cygb‐SH). This protein was run through a G‐25 column to remove the excess DTT then aliquoted for treatment to modify the free thiol groups as follows: reaction with 2 mm NEM (C_6_H_7_NO_2_ MW: 125.13) to alkylate reactive thiol groups (Cygb‐SC), or 4 mm 4,4′‐dithiodipyridine (4‐PDS, C_10_H_8_N_2_S_2_ MW: 220.3) to drive disulfide bond formation (Cygb‐SS) at room temperature for 1 h. After the incubation period, the samples were concentrated and again run through a G‐25 column to remove excess reactants. The purity of the three products Cygb‐SS, Cygb‐SH, and Cygb‐SC was determined by comparing the concentration of a given sample of Cygb (measured by the pyridine hemochromagen assay 26) with the concentration of free thiols of the same Cygb sample (measured by the 4‐PDS method 27). The final average percentage of thiol modification is > 90%.

### PAGE and Coomassie staining of pure Cygb

Pure Cygb (10 ng) was loaded to 4–20% gradient gels under nonreducing conditions. Protein samples were separated at 150 V for ~ 90 min. At the end of separation, gels were incubated with 1% Coomassie stain in 40% methanol and 10% acetic acid. After 2 h of staining, gels were destained with repeated 15 min incubations with ~ 20 mL of destaining solution (40% methanol and 10% acetic acid) until bands were clear. Gels were then imaged with a Bio‐Rad (Hercules, CA, USA) Versadoc imaging system using quantityone imaging software.

### Measurements of O_2_ binding and redox state of Cygb(Fe^2+^)

A Cary 50 UV/Vis spectrophotometer was used to measure the changes in absorbance at 428 nm (A_428_; peak absorbance of Cygb(Fe^2+^)) with time in the process of reduction of Cygb(Fe^3+^) and O_2_ binding to Cygb(Fe^2+^). Simultaneously, a Clark O_2_ electrode was placed in the solution to monitor the changes in O_2_ concentration ([O_2_], 37 °C). Both A_428_ and [O_2_] were sampled at the same rate (10 readings per second). After 1.5 mL of buffer solution and Cygb(Fe^3+^) were added into the cuvette, the cuvette was covered with a Parafilm membrane. An argon gas tube with an outer diameter of 1 mm was inserted into the cuvette that was covered by a parafilm membrane and positioned above the solution surface to keep an argon flow in the cuvette to gradually remove O_2_ from the solution. When O_2_ in the solution decreased by about half, ~ 1 mm dithionite (DT) was injected into the solution to rapidly scavenge all O_2_ and reduce Cygb(Fe^3+^) to Cygb(Fe^2+^) in the cuvette. Although the recordings of O_2_ concentration and the absorbance of Cygb(Fe^2+^) were started before injection of DT, time 0 was assigned to the time point at which DT was added into the solution. At this moment, both [O_2_] and A_428_ suddenly changed. This time point was used as the starting point for synchronizing the readings of A_428_ and [O_2_], which is important for matching the percent of O_2_ bound to Cygb(Fe^2+^) with the corresponding [O_2_] in the solution. After Cygb was reduced and O_2_ was scavenged from both the solution and the gas phase, the gas tube was taken out of the cuvette, and the hole in the Parafilm membrane for the gas tube was sealed by a piece of Parafilm. A syringe needle was used to make a tiny hole on the Parafilm so that O_2_ in the air could enter the gas phase of the cuvette slowly. The entered O_2_ was gradually dissolved into the test solution to raise the O_2_ concentration in the solution at a rate of ~ 0.04–0.06 μm·s^−1^ to maintain the O_2_ binding process near the equilibrium. The dissolved O_2_ can bind on Cygb(Fe^2+^) to form Cygb(Fe^2+^O_2_).

### Electrochemical measurements of the rate of O_2_‐dependent NO metabolism by Cygb

The measurements were performed in a four‐port water‐jacketed electrochemical chamber (NOCHM‐4 from WPI, Sarasota, FL, USA) containing 2 mL of Dulbecco's PBS (Thermo Scientific, South Logan, UT, USA) as previously described 5, 19. The NO solution was also prepared as previously described 28, 29. After the NO and O_2_ electrodes had stabilized, NO (0.5 μm) was injected into the aerated buffer solution (under room air) in the absence of Cygb and reductants. When the NO concentration decreased to baseline, 0.4 μm Cygb, 400 U·mL^−1^ superoxide dismutase (SOD) from horseradish (Sigma‐Aldrich, St. Louis, MO, USA) or 50 μm SOD mimetic (GC4419, Galera Therapeutics Inc., Malvern, PA, USA), and either ascorbate (Asc; Sigma‐Aldrich) or cytochrome b5 reductase (b5R) with NADH (Sigma‐Aldrich) and cytochrome b5 (b5) were added to the chamber. Then 0.5 μm NO was injected into the solution to measure the rate of NO consumption under room air. To measure the rate of NO consumption by Cygb at different O_2_ concentrations below 200 μm, argon gas tube was introduced into the chamber above the solution through a hole in the cap for removing O_2_ from the solution. While [O_2_] in the solution was gradually decreased due to the flow of argon gas, equal amounts of 0.5 μm NO were repeatedly injected into the solution. Changes in [O_2_] and [NO] over time were recorded by the O_2_ and NO electrodes, respectively. From the recorded temporal [NO] and [O_2_] curves, the rate of NO decay (V_NO_) at each NO peak and the corresponding [O_2_] were measured and used to plot the V_NO_ vs. [O_2_] curves.

### Spectrophotometric measurements of Cygb(Fe^3+^) reduction by Asc or by the b5R/b5/NADH enzyme‐reducing system

Measurements were performed on a Cary 50 UV/Vis spectrophotometer. After 1.5 mL of buffer solution was added into the cuvette, the cuvette was covered with a Parafilm membrane. A Clark O_2_ electrode was placed in the solution to monitor O_2_ concentration. The measured O_2_ concentrations were recorded by an Apollo 4000 Free Radical Analyzer (WPI) using a Clark electrode. The solution was stirred using a magnetic stirring bar that was placed on the bottom of the cuvette. An argon gas tube was inserted in the cuvette to bubble argon into the solution for 15 min to quickly remove O_2_. Before injecting Cygb (for reduction by Asc) or Cygb + b5 + NADH (for reduction by the reducing system b5R/b5/NADH) into the solution, the argon gas tube was placed just above the solution surface to keep an argon flow in the cuvette. About 20 min after injection of Cygb or Cygb/b5/NADH, Asc or b5R was added to the solution to initiate reduction of Cygb(Fe^3+^), respectively. The reaction process was monitored by measuring the changes in absorbance at 416 nm (peak absorbance of Cygb(Fe^3+^)) with time 30.

### Equations for determining rate constants of Cygb reduction

The reduction scheme of Cygb(Fe^3+^) reduction by Asc has been proposed in our previous paper 30:

(1) $$A+B\overset{k_{1}}{\underset{k_{1}}{⇌}}C\overset{k_{2}}{\underset{k_{2}}{⇌}}D$$

where *A* is reductant, *B* is Cygb(Fe^3+^), *C* is the complex Cygb(Fe^3+^ *A*), and *D* is Cygb(Fe^2+^ *A* ^+^). In the following derivation process, concentrations of *A*,* B*,* C*,* D* and the total Cygb protein *E* are presented by [*A*], [*B*], [*C*], [*D*], and [*E*], respectively. [*E*] = [*B*] + [*C*] + [*D*] for any time *t*. Since *E* is the total Cygb concentration including *B*,* C*, and *D*,* E* is considered as a constant in the experiments assuming that Cygb only has the three forms *B*,* C*, and *D*. Concentrations of *A*,* B*,* C*, and *D* in the beginning of the reaction (*t = *0) are written as [*A*]_0_, [*B*]_0_, [*C*]_0_, [*D*]_0_, respectively. At *t *= 0, we assume that [*B*]_0_ = [*E*], [*C*]_0 _= [*D*]_0 _= 0. Using steady‐state approximation, the complex *C* is an intermediate and its concentration is assumed in the steady‐state or *d*[*C*]*/dt = *0. Thus, we can obtain:

$$\begin{array}{cc} \frac{d[B]}{dt} & =-k_{1}\left[A\right]\left[B\right]+k_{-1}\left[C\right],\\ \frac{d[C]}{dt} & =k_{1}\left[A\right]\left[B\right]+k_{-2}\left[D\right]-\left(k_{-1}+k_{2}\right)\left[C\right]=0,\\ \;[B]+[C]+[D] & =\left[E\right]\;\text{and}\;\frac{d[B]}{dt}=-\frac{d[D]}{dt} \end{array}$$

Under the steady‐state approximation (*dC/dt *= 0), the rate of *B* consumption is equal to the rate of *D* formation. From the above equations we can obtain (see Data S1):

(2) $$\frac{d[B]}{dt}=\frac{k_{-1}k_{-2}\left[E\right]}{k_{-1}+k_{2}+k_{-2}}-\left(\frac{k_{-1}k_{-2}+k_{1}\left(k_{2}+k_{-2}\right)\left[A\right]}{k_{-1}+k_{2}+k_{-2}}\right)\left[B\right]=-g_{1}\left(\left[B\right]-\left[B_{b}\right]\right)$$

where *g*_1_ can be considered as the rate constant of reduction of *B* by *A*. In Eqn (3), *d*[*B*]*/dt* approaches 0 as *t* approaches to infinity. Thus, [*B*] approaches [*B*_b_] as *t* approaches infinity. Here, [*B*_b_] is concentration of *B* at *t *= ∞. From Eqn (3) we obtain:

(3) $$\text{ln}\left(\left[B\right]-\left[B_{b}\right]\right)=-g_{1}t+g_{0}$$

where *g*_0_ is an integration constant. In experiments for measuring the reduction of Cygb(Fe^3+^), we used a UV/Vis spectrophotometer to monitor the changes in absorbance (Abs) at wavelength 416 nm. According to the Beer–Lambert Law, we have:

(4) $$\text{Abs}=\varepsilon_{B}\left[B\right]+\varepsilon_{C}\left[C\right]+\varepsilon_{D}\left[D\right]$$

where ε_*B*_, ε_*C*_ and ε_*D*_ are the molar extinction coefficients of *B*,* C* and *D*, respectively. Abs is the absorbance at time *t*. If time *t* is large (theoretically *t* approaches infinity), Eqn (4) can be written in the following form:

(5) $$\text{Abs}{}_{b}=\varepsilon_{B}\left[B_{b}\right]+\varepsilon_{C}\left[C_{b}\right]+\varepsilon_{D}\left[D_{b}\right]$$

where Abs_b_ is the aborbance as *t* approaches infinity.

We also have: [*D*] = [*E*] − [*B*] − [*C*] and [*D*_b_] = [*E*] − [*B*_b_] − [*C*_b_]. Here, [*C*_b_] and [*D*_b_] are concentrations of *C* and *D* as *t* approaches infinity. Combining these two equations with Eqs (4) and (5) gives:

(6) $$\text{Abs}=\left(\varepsilon_{B}-\varepsilon_{D}\right)\left[B\right]+\left(\varepsilon_{C}-\varepsilon_{D}\right)\left[C\right]+\varepsilon_{D}\left[E\right]$$

(7) $$\text{Abs}{}_{b}=\left(\varepsilon_{B}-\varepsilon_{D}\right)\left[B_{b}\right]+\left(\varepsilon_{C}-\varepsilon_{D}\right)\left[C_{b}\right]+\varepsilon_{D}\left[E_{b}\right]$$

Then we can get:

(8) $$\text{Abs}-{\text{Abs}}_{b}=\left(\varepsilon_{B}-\varepsilon_{D}\right)\left(\left[B\right]-\left[B_{b}\right]\right)+\left(\varepsilon_{C}-\varepsilon_{D}\right)\left(\left[C\right]-\left[C_{b}\right]\right)$$

Under steady‐state approximation, *C* is a constant during the measurements of absorbance. Thus, we can obtain:

(9) $$\left[B\right]-\left[B_{b}\right]=\frac{\text{Abs}-{\text{Abs}}_{b}}{\varepsilon_{B}-\varepsilon_{D}}$$

Substituting Eqn (9) into Eqn (3), we have:

(10) $$\text{ln}\left(\text{Abs}-{\text{Abs}}_{b}\right)=-g_{1}t+g_{0}+\text{ln}\left(\varepsilon_{B}-\varepsilon_{D}\right)=-g_{1}t+g$$

Equation (10) indicates that the plot of ln(Abs *− *Abs_b_) vs. *t* from experimental data will generate a straight line with a slope of −*g*_1_.

## Results

### PAGE and Coomassie staining of Cygb

**Figure 1:**
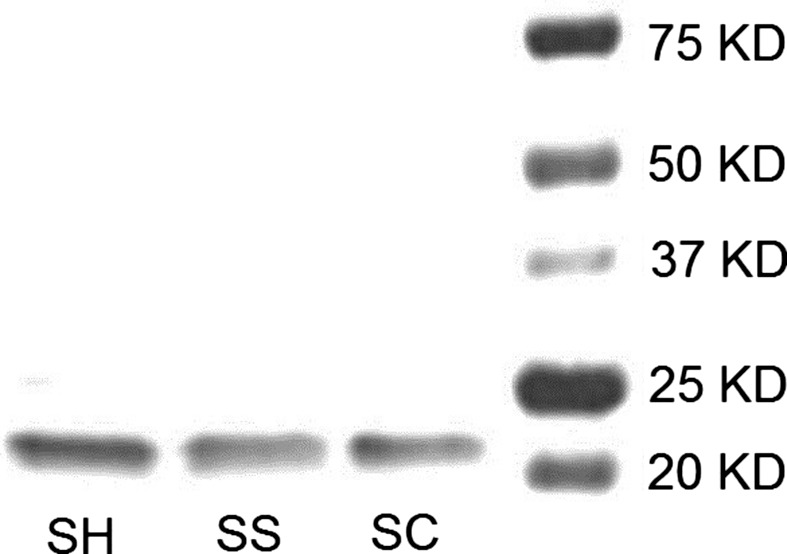
SDS/PAGE analysis of the Cygb preparations (SH, SS and SN represent Cygb‐SH, Cygb‐SS, and Cygb‐SC). Cygb (10 ng) was loaded on PAGE gels under nonreducing conditions. Gels were Coomassie‐stained and imaged. A single band is seen at ~ 21 kDa indicating that only monomeric Cygb was present.

PAGE followed by Coomassie staining was performed to characterize each of the Cygb preparations including the reduced protein Cygb‐SH (SH), the oxidized disulfide Cygb‐SS (SS), and NEM‐modified Cygb‐SC (SC). The resolved gel of the three types of Cygb preparations is shown in Fig. 1. The pure Cygb bands appear with a molecular weight of ~ 21 kDa. The bands in the right column are protein markers with molecular weights indicated. In all three of the Cygb preparations, only monomeric Cygb is present. Any possible dimer form may have been converted into monomer in the preparation of Cygb samples with DTT.

### O_2_‐binding curves of modified Cygbs

**Figure 2:**
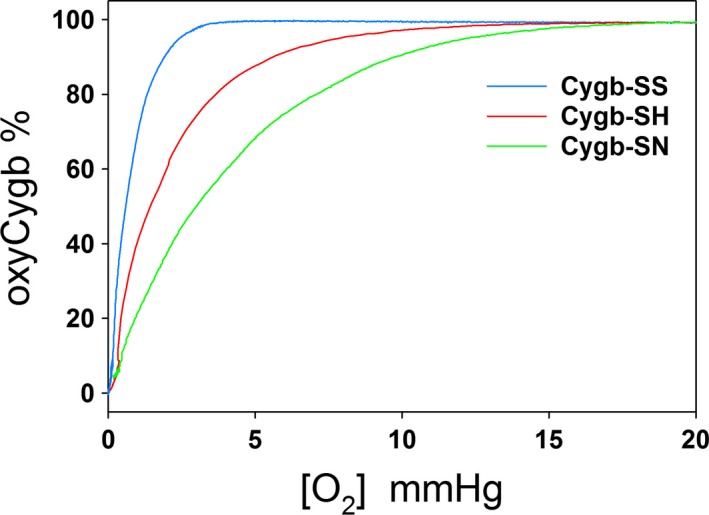
Experimental curves of O_2_ binding to 3 μm Cygb‐SS, Cygb‐SH, and Cygb‐SC.

To examine the effect of modifying the sulfhydryl groups of cysteine on the affinity of O_2_ binding to Cygbs, we measured O_2_‐binding curves for each of the three Cygb preparations at 37 °C. Figure 2 shows typical experimental O_2_‐binding curves. It can be seen that the P_50_ of Cygb has the following order: Cygb‐SS < Cygb‐SH < Cygb‐SC. The mean and standard errors of the P_50_ values (*n* = 3) for the three Cygbs are listed in Table 1 (unit in mmHg or Torr).

**Table 1:** Measured P_50_ (mmHg) for O_2_ binding to Cygb

Proteins	Cygb‐SS	Cygb‐SH	Cygb‐SC
Mean	0.7	1.4	2.9
SE	0.1	0.1	0.2

### Reduction of modified Cygbs

**Figure 3:**
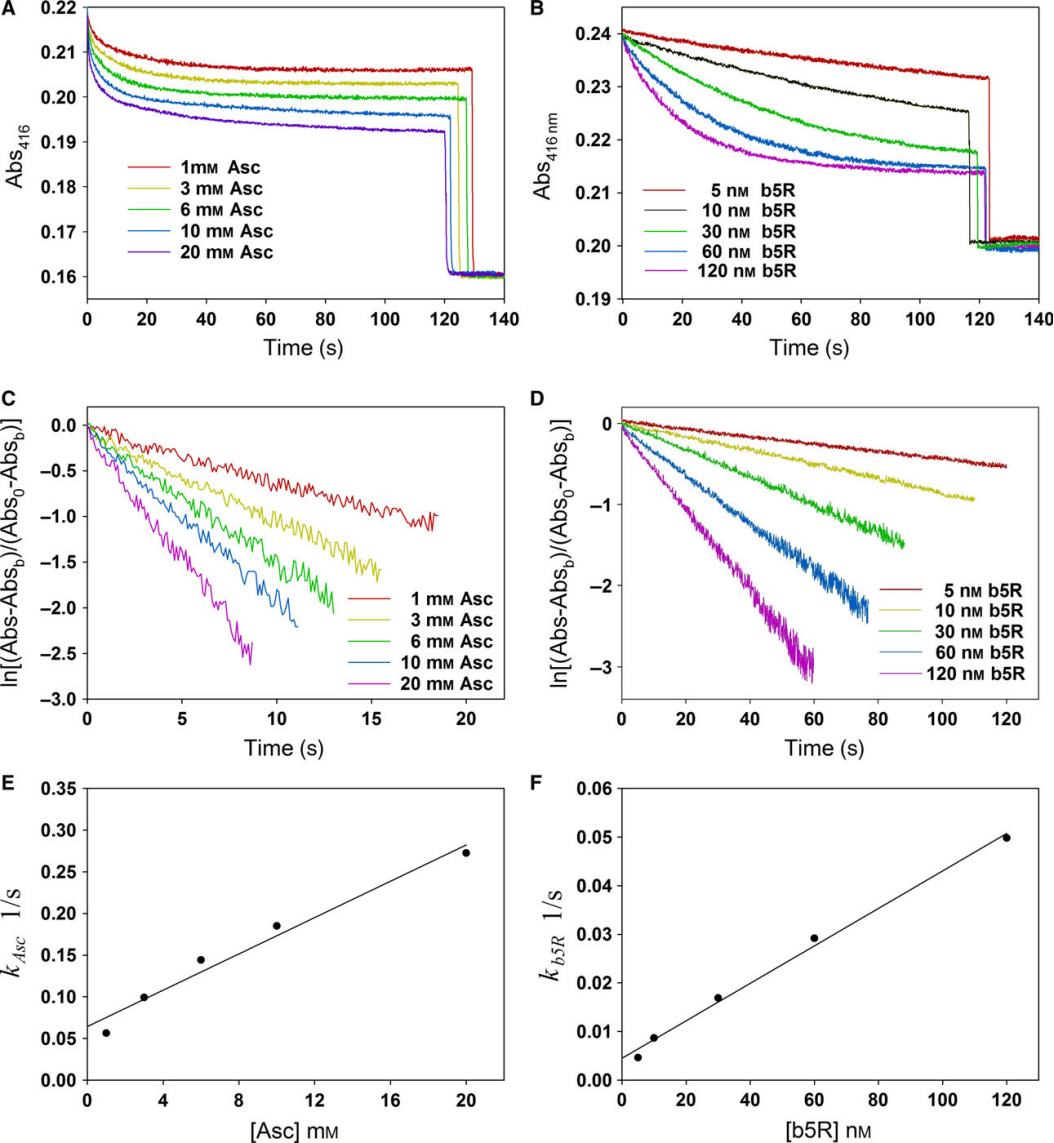
Reduction of Cygb‐SH by cellular reductants. Measurements of absorbance at 416 nm while 2 μm Cygb‐SH was reduced by different concentrations of Asc (A) or by different concentrations of b5R in the presence of 0.5 μm b5 and 100 μm NADH (B). Plot of ln(Abs − Abs_b_) vs. time as 2 μm Cygb‐SH was reduced by varied concentrations of Asc (C), and by varied concentrations of b5R in the presence of 0.5 μm b5 and 100 μm NADH (D). Plots of rate constants of Cygb‐SH reduction vs. Asc (E) or b5R (F). Rate constants of Cygb‐SH reduction by varied Asc or b5R were obtained from the slopes of the lines in (C) and (D).

**Figure 4:**
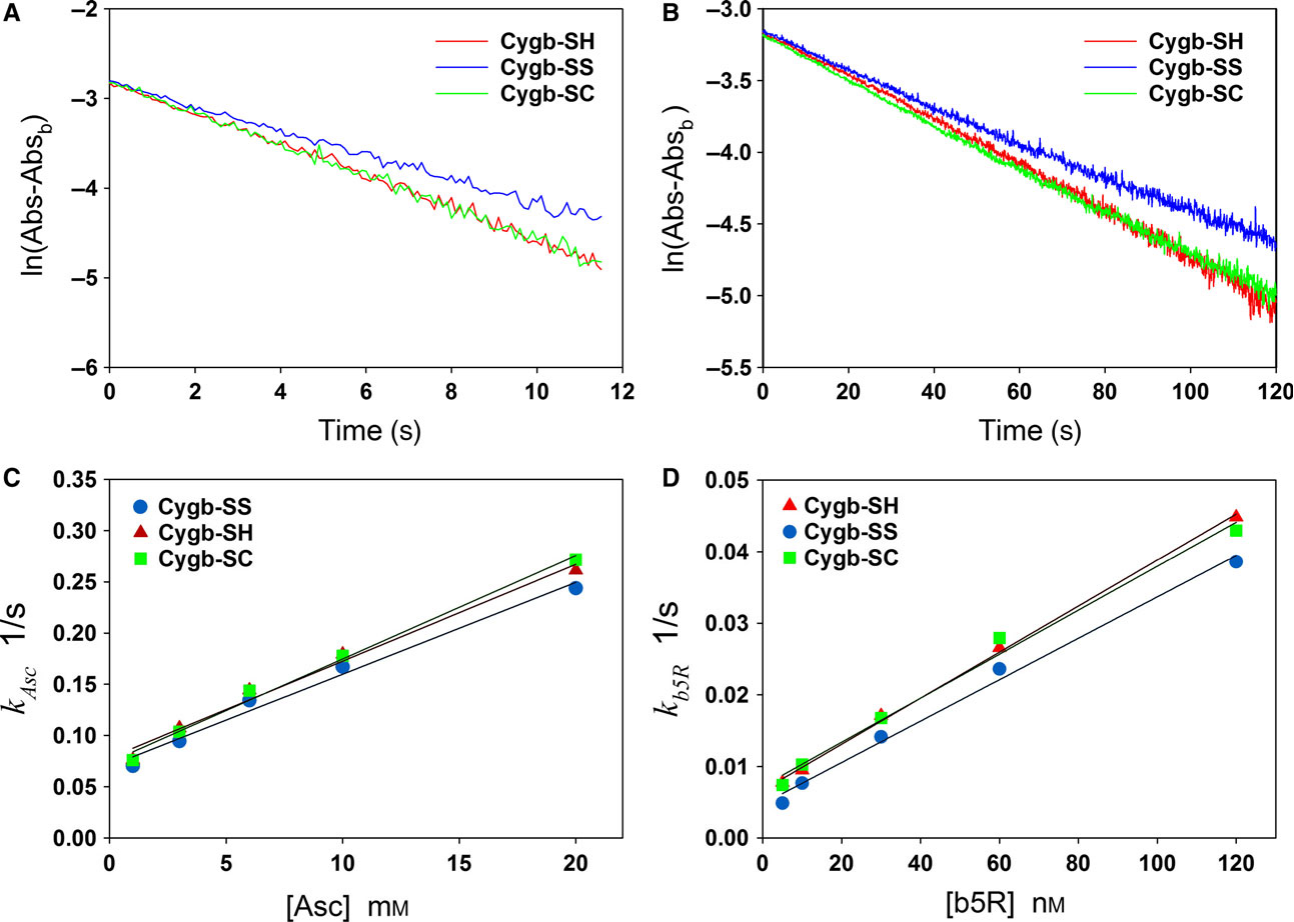
Reduction of three Cygbs (Cygb‐SH, Cygb‐SS, and Cygb‐SC) by varied concentrations of Asc or b5R. Plots of ln(Abs − Abs_b_) vs. time as the three Cygbs (~ 3 μm) were reduced by 10 mm Asc (A) or 30 nm b5R in the presence of 0.5 μm b5 and 100 μm NADH (B). Plots of rate constants of 2 μm Cygbs (Cygb‐SH, Cygb‐SS, and Cygb‐SC) reduction by varied Asc concentrations (C) or by varied b5R concentration in the presence of 0.5 μm b5 and 100 μm NADH (D).

During NO dioxygenation, O_2_ binds to Cygb(Fe^2+^) to form Cygb(Fe^2+^O_2_) which can be oxidized to Cygb(Fe^3+^) by NO. To maintain continuity of NO dioxygenation, a reductant (such as Asc) is required for reducing Cygb(Fe^3+^) back to Cygb(Fe^2+^). The reduction of the three Cygb(Fe^3+^) preparations by Asc (1–20 mm) and by b5R (5–120 nm)/b5 (0.5 μm)/NADH (100 μm) was measured with a spectrophotometric assay. It was observed that either Asc or b5R in the presence of b5 (0.5 μm) and NADH (100 μm) cannot fully reduce each of the Cygbs including Cygb‐SH, Cygb‐SC, and Cygb‐SS in the concentration range listed above, but full reduction could be accomplished by 1 mm DT. In Fig. 3 we demonstrated the typical experimental curves for reduction of Cygb‐SH by Asc (Fig. 3A) and b5R/b5/NADH (Fig. 3B). From the experimental data, we plotted ln(Abs − Abs_b_) vs. time *t* based on Eqn (10) (Fig. 3C,D). All plots with Asc as the reductant are linear with time after 2–5 s from the initial injection of Asc (Fig. 3C). From the slopes of these lines, we obtained the pseudo first‐order rate constant *g*_1_, which (shown as *k*_Asc_ or *k*_b5R_ in Fig. 3E–F) is linear with reductant concentration [Asc] or [b5R]. In Fig 4A,B, we demonstrated the plots of ln(Abs − Abs_b_) vs. time *t* for Cygb‐SH, Cygb‐SS, and Cygb‐SC reduction by 10 mm Asc or 30 nm b5R in the presence of 0.5 μm b5 and 100 μm NADH. All plots are nearly linear. From the slopes of the lines, we can determine the pseudo first‐order rate constants of reduction of the three Cygbs by Asc (10 mm) or by b5R (30 nm) in the presence of 0.5 μm b5 and 100 μm NADH. The pseudo first‐order rate constants of Cygb‐SH, Cygb‐SS, and Cygb‐SC at other Asc and b5R concentrations are shown in Fig 4C (for Asc) and Fig. 4D (for b5R).

### O_2_‐dependent NO metabolism by modified Cygbs

**Figure 5:**
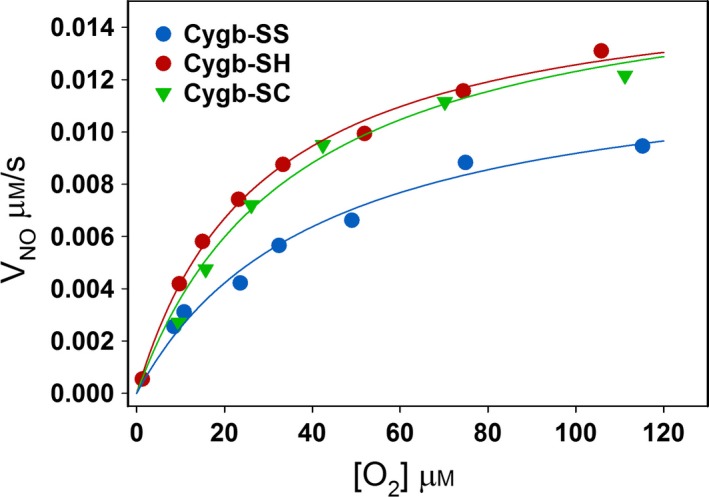
Plots of V_NO_ of Cygbs vs [O_2_]. Rate of NO metabolism by 0.4 μm of Cygb‐SS, Cygb‐SH, and Cygb‐SC in the presence of 400 μm Asc at different O_2_ concentrations.

The Cygb‐mediated O_2_‐dependent NO metabolism was examined by simultaneously measuring changes in NO and O_2_ concentrations with NO and O_2_ electrodes. The rates of NO metabolism in the presence of 0.4 μm Cygbs and 400 μm Asc under varying O_2_ concentrations are shown in Fig. 5. The rate of NO metabolism by Cygb‐SS under room air is lower than that by Cygb‐SH and Cygb‐SC. The measured rates of NO decay (V_NO_) under room air (at ~ 200 μm [O_2_]) are 13.0 ± 1.2 nm·s^−1^ (Cygb‐SS), 16.1 ± 1.0 nm·s^−1^ (Cygb‐SH), and 16.7 ± 1.2 nm·s^−1^ (Cygb‐SC). When O_2_ in the solution was gradually decreased, the rate of NO metabolism by the three Cygbs gradually decreased in a similar pattern.

## Discussion

Like other globins, Cygb binds O_2_ at the sixth coordination site apical to the heme iron. The intrinsic histidine of Cygb competes with O_2_ binding at the sixth coordination site. It has been reported that formation of an intramolecular disulfide bond in Cygb can largely change the equilibrium binding constant of the intrinsic distal histidine 21, which can significantly change the apparent O_2_‐binding equilibrium constant. The disulfide bond, if formed, can be reduced by DTT to form two free SH groups. It was reported that the P_50_ of O_2_ binding to Cygb is 1.8 Torr after Cygb is treated with DTT 20, 31, while the P_50_ of O_2_ binding to Cygb without DTT treatment was reported in the range between 0.2 and 1 Torr 3, 20, 32. We tested O_2_ binding behaviors for Cygb‐SS (P_50_ = 0.7 Torr), Cygb‐SH (P_50_ = 1.4 Torr) and Cygb‐SC (P_50_ = 2.9 Torr). The measured P_50_ values for Cygb‐SS and Cygb‐SH (Fig. 2 and Table 1) are in the range of reported values in the literature. Furthermore, we observed that P_50_ for Cygb‐SC is greater than that for Cygb‐SH.

We then examined the effect of modification of the sulfhydryl group of the cysteines of Cygb on the rate of Cygb reduction. Chemical kinetics with mathematical modeling is a powerful tool to study complicated reaction processes 5, 33, 34, 35. Using this tool, we obtained Eqn (10) for determining the rate constant of Cygb reduction from experimental data of spectrophotometric measurements. It was observed that the rate of Cygb‐SH, Cygb‐SC, and Cygb‐SS reduction by Asc and by b5R/b5/NADH increases with Asc and b5R concentration, respectively. A group of typical experimental curves for the reduction of Cygb‐SH with varying concentrations of Asc (1–20 mm) and b5R (5–120 nm) were shown in Fig. 3A,B. Using Eqn (10), we determined the pseudo first‐order rate constants from the experimental curves in Fig. 3A,B. The data process was demonstrated in Fig. 3C–F. From Fig. 3E,F, we can see that the determined pseudo first‐order rate constants are nearly linear with Asc or b5R concentrations, and the intercepts of the fitted lines are greater than zero. From the slopes of the plots shown in Fig. 4A–D, we can see that the pseudo first‐order rate constants of Cygb‐SH, Cygb‐SC, and Cygb‐SS reduction by Asc and b5R/b5/NADH are close to each other with the rate constant of Cygb‐SS reduction ~ 25% lower than those of Cygb‐SH and Cygb‐SC. Because the rate of Cygb reduction is the rate‐limiting step for NO dioxygenase activity of Cygb 23, the small differences (~ 25%) in rate constants of Cygb reduction after modification of sulfhydryl groups imply that the modification of sulfhydryl groups on the Cygb only cause a small effect on the NO dioxygenase activity of Cygb although this modification more largely shifts the apparent O_2_‐binding constant. Correspondingly, the differences in the O_2_‐dependent NO dioxygenase activity for the three Cygbs (Fig. 5) parallel the differences in their reduction rates with ~ 25% decrease seen in Cygb‐SS compared to Cygb‐SH and Cygb‐SC (Fig. 4A).

The process of NO dioxygenation requires O_2_ binding to Cygb to form Cygb(Fe^2+^O_2_) which can rapidly react with NO. If we gradually remove O_2_ from the test solution, O_2_ concentration in the solution will gradually decrease. Since the modification of sulfhydryl groups of Cygb can shift the P_50_, differences in the Cygb(Fe^2+^O_2_) concentration for the three Cygb preparations may be seen when pO_2_ drops below 10 Torr (Fig. 2). From Fig. 2 we see that Cygb‐SS will have the highest Cygb(Fe^2+^O_2_) concentration and Cygb‐SC will have the lowest. Thus, under low pO_2_ conditions, one could speculate that the NO dioxygenase activity by Cygb‐SS might be the highest among the three Cygbs. However, only a small change in the NO dioxygenase activity was observed for Cygb‐SS compared to Cygb‐SH or Cygb‐SC at O_2_ concentrations below 120 μm (Fig. 5). Thus, although the modification of the sulfhydryl groups on the Cygb can largely increase the apparent O_2_‐binding constant of Cygbs in the absence of NO, the bound O_2_ on ferrous Cygb or Cygb(Fe^2+^O_2_) can be immediately removed by NO if NO is present in the solution because NO rapidly reacts with Cygb(Fe^2+^O_2_) to form ${\text{NO}}_{3}^{-}$ and Cygb(Fe^3+^). As a result, almost all of Cygb(Fe^2+^O_2_) will react with NO in a short time. The NO dioxygenase activity is then limited by the rate‐limiting step, reduction of Cygb. The modification of the sulfhydryl groups on Cygb does not greatly change the rate of Cygb reduction; therefore, the NO dioxygenase activity is only modestly affected by the modification of the sulfhydryl groups of Cygb.

In summary, the modification of the sulfhydryl groups on Cygb can largely change (~ 4‐fold) the apparent constant of O_2_ binding to Cygb. However, this modification only causes a small difference (~ 25%) in the rate constant of Cygb reduction and NO consumption by Cygb. These results indicate that it is possible to largely change one property of Cygb (such as O_2_ binding ability) and keep another property of Cygb (such as NO dioxygenase activity) almost unchanged or only modest change.

## Author contributions

XL and JLZ supervised the work of this study. AL provided supervision and support for DZ. XL wrote the paper with input from JLZ. DZ performed spectrophotometric and electrochemical experiments. XL and DZ designed experiments and analyzed data with input from JLZ. CH and DZ prepared Cygb samples and wrote the related methods. JB performed PAGE experiments and wrote the related methods. XL, JLZ, CH, JB, DZ, and AL all contributed to the manuscript and its revisions.

## Supporting information


**Data S1.** Equations for determining rate constants of Cygb reduction.
