# Supplementary material for: Oxygen binding and nitric oxide dioxygenase activity of cytoglobin are altered to different extents by cysteine modification
Source: FEBS Open Bio. 2017 May 18;7(6):845–53. doi: 10.1002/2211-5463.12230 (PMC5458454; doi:10.1002/2211-5463.12230)
Supplement: Supplementary file 1 — Data S1. Equations for determining rate constants of Cygb reduction. [file FEB4-7-845-s001.pdf]

Equations for Determining Rate Constants of Cygb Reduction

The reduction scheme of Cygb(Fe^{3+}) reduction by Asc has been proposed in our previous paper:

$$A + B \underset{k_{-1}}{\overset{k_1}{\rightleftharpoons}} C \underset{k_{-2}}{\overset{k_2}{\rightleftharpoons}} D \qquad (1)$$

where A is reductant, B is Cygb(Fe^{3+}), C is the complex Cygb($Fe^{3+}A$), and D is Cygb($Fe^{2+}A^{+}$). The rate equations for B and C can be written in the following form:

$$\frac{d[B]}{dt} = -k_1[A][B] + k_{-1}[C] \qquad (2)$$

$$\frac{d[C]}{dt} = k_1[A][B] + k_{-2}[D] - (k_{-1} + k_2)[C] \qquad (3)$$

$$[B] + [C] + [D] = [E] \qquad (4)$$

At t=0, we assume that B_0=E, C_0=D_0=0, where B_0, C_0 and D_0 are the initial concentration of B, C and D, respectively. E is the total Cygb concentration including B, C and D. Using steady-state approximate, the complex C is an intermediate and its concentration is assumed in the steady-state or $dc/dt \approx 0$. Thus we have the following equation from Eq (3):

$$[C] = \frac{k_1[A][B] + k_{-2}[D]}{k_{-1} + k_2} \qquad (5)$$

From Eq (4) we have:

$$[D] = [E] - [B] - [C] \qquad (6)$$

Substitution of Eq (6) into (5) gives:

$$[C] = \frac{k_1[A][B] + k_{-2}([E] - [B] - [C])}{k_{-1} + k_2}$$

$$[C] = \frac{k_{-2}[E] + (k_1[A] - k_{-2})[B]}{k_{-1} + k_2 + k_{-2}} \qquad (7)$$

Substitution of Eq. (7) into Eq. (6) gives:

$$\begin{aligned}\frac{d[B]}{dt} &= -k_1[A][B] + k_{-1}\frac{k_{-2}[E] + (k_1[A] - k_{-2})[B]}{k_{-1} + k_2 + k_{-2}} \\ &= \frac{-k_1(k_{-1} + k_2 + k_{-2})[A][B] + k_{-1}k_{-2}[E] + k_{-1}(k_1[A] - k_{-2})[B]}{k_{-1} + k_2 + k_{-2}} \\ &= \frac{k_{-1}k_{-2}[E] - k_1(k_2 + k_{-2})[A][B] - k_{-1}k_{-2}[B]}{k_{-1} + k_2 + k_{-2}} \\ &= \frac{k_{-1}k_{-2}}{k_{-1} + k_2 + k_{-2}}[E] - \frac{k_{-1}k_{-2} + k_1(k_2 + k_{-2})[A]}{k_{-1} + k_2 + k_{-2}}[B]\end{aligned} \qquad (8)$$

At t=0, B=B_0=E. Dividing both sides of Eq. (8) by B_0 or E, Eq. (8) can be simply written in the following form:

$$\frac{d[B]}{dt} = g_1([B_b]-[B]) = -g_1([B]-[B_b]) \quad (9)$$

where g_1 can be considered as the pseudo first order rate constant of reduction of B by A.

$$g_1 = \frac{k_{-1}k_{-2} + k_1(k_2 + k_{-2})[A]}{k_{-1} + k_2 + k_{-2}} \quad (10)$$

In Eq. (9), dB/dt approaches to 0 as t approaches to infinity. Thus B approaches B_∞ as t approaches infinity. From Eq. (9) we can obtain:

$$\ln([B]-[B_b]) = -g_1 t + g_0 \quad (11)$$

In experiments for measuring the reduction of Cygb(Fe^{3+}) by a reductant, we used a UV/Vis spectrophotometer to monitor the changes in absorbance at wavelength 416 nm. According to the Beer-Lambert Law, we have:

$$Abs = \varepsilon lc \quad (12)$$

Where A is the absorbance at a given wavelength, ε is the molar extinction coefficients at the given wavelength, l is the length of the light path, and c is the concentration of the solution of the sample used in the experiment. Considering that l is 1 cm in our experiments, we have the following equation for changes in the absorbance of solution while Cygb(Fe^{3+}) is reduced by Asc or CBR/B5/NADH reductant system:

$$[A] = \varepsilon_B[B] + \varepsilon_C[C] + \varepsilon_D[D] \quad (13)$$

where ε_B, ε_C and ε_D are the molar extinction coefficients of B, C and D, respectively. A is the absorbance at time t. If time t is very big (theoretically t approaches to infinity), Eq. (13) can be written in the following form:

$$[A]_\infty = \varepsilon_B[B_b] + \varepsilon_C[C_b] + \varepsilon_D[D_b] \quad (14)$$

From Eq. (4) we have: $[D]=[E]-[B]-[C]$ and $[D_b]=[E]-[B_b]-[C_b]$. Combination of the two equations with Eqs. (13) and (14) gives:

$$[A] = (\varepsilon_B - \varepsilon_D)[B] + (\varepsilon_C - \varepsilon_D)[C] + \varepsilon_D[E] \quad (15)$$

$$[A_b] = (\varepsilon_B - \varepsilon_D)[B_b] + (\varepsilon_C - \varepsilon_D)[C_b] + \varepsilon_D[E_b] \quad (16)$$

Then we can get:

$$[A]-[A_b] = (\varepsilon_B - \varepsilon_D)([B]-[B_b]) + (\varepsilon_C - \varepsilon_D)([C]-[C_b]) \quad (17)$$

Under steady-state approximation, C is a constant during the measurements of absorbance. Thus Eq. (17) can be simplified as the following form:

$$[B]-[B_b] = \frac{[A]-[A_b]}{\varepsilon_B - \varepsilon_D} \quad (18)$$

Substituting Eq. (18) into Eq. (11), we have:

$$\ln([A]-[A_b]) = -g_1 t + g_0 + \ln(\varepsilon_B - \varepsilon_D) = -g_1 t + g \quad (19)$$
